# Bioinspired 3D Printing of Lignocellulose-Based Multimaterial Composites for Extracellular Matrix-Mimicking Architectures

**DOI:** 10.3390/biomimetics11060429

**Published:** 2026-06-16

**Authors:** Youjin Seol, Myoung Joon Jeon, Sayan Deb Dutta, Youjin Jeong, Ki-Taek Lim

**Affiliations:** 1 Department of Biosystems Engineering, Kangwon National University, Chuncheon-si 24341, Gangwon-do, Republic of Korea; 2 Interdisciplinary Program in Smart Agriculture, Kangwon National University, Chuncheon-si 24341, Gangwon-do, Republic of Korea; 3 Institute of Forest Science, Kangwon National University, Chuncheon-si 24341, Gangwon-do, Republic of Korea

**Keywords:** ECM heterogeneity, bioinspired scaffolds, multimaterial printing, lignocellulose composites, tissue-engineering applications

## Abstract

The extracellular matrix (ECM) provides a dynamic microenvironment that regulates cell proliferation, migration, and tissue remodeling during wound healing. However, replicating the structural and functional complexity and ECM heterogeneity of native skin ECM remains challenging with conventional single-material hydrogels. Recent advances in multimaterial 3D bioprinting have enabled the spatial integration of diverse biomaterials within a single construct. Lignocellulose has attracted increasing attention as a promising biomaterial for recreating key structural features of the native ECM because of its fibrous architecture, mechanical strength, and biocompatibility. This review offers a comprehensive and integrated perspective on the use of lignocellulose-based multimaterial printing to recreate ECM-mimicking architectures, an underexplored area at the intersection of biomaterials and biofabrication. The roles of cellulose, hemicellulose, and lignin in printability, scaffold stability, porosity, bioactivity, and wound-healing performance are discussed. Representative studies have demonstrated that lignocellulose-based multimaterial bioinks provide porous architectures that support cell adhesion, proliferation, and tissue regeneration. These benefits are accompanied by improved mechanical performance, as cellulose nanofibers exhibit elastic moduli exceeding 100 GPa, and lignin-containing hydrogels have achieved compressive moduli of up to 135 kPa. Such mechanical advantages make lignocellulosic materials particularly attractive for fabricating ECM-mimicking scaffolds that require long-term structural integrity. Finally, key design considerations and current limitations associated with lignocellulose-based multimaterial bioprinting are critically discussed. A framework for the rational design of lignocellulose-based multimaterial bioinks is presented, together with future directions toward gradient and adaptive scaffolds, smart wound dressings, and advanced wound-healing applications.

## 1. Introduction

The extracellular matrix (ECM) provides a dynamic microenvironment that regulates cell proliferation, migration, differentiation, and tissue remodeling during wound healing [1,2]. The structure and function of the ECM are dynamic, as reflected in processes such as fibrin clot formation, regulation of inflammatory responses, and continuous matrix remodeling [3,4,5]. Although electrospun nanofibers and self-assembled peptide hydrogels can reproduce certain structural features of the native ECM, they remain limited in their ability to replicate its hierarchical organization and multifunctionality [6,7,8]. By contrast, 3D bioprinting enables the fabrication of constructs with controlled architectures and spatially defined material distributions, allowing more precise reproduction of tissue-specific microenvironments [9,10,11]. 3D-printed hydrogels have been widely adopted in multimaterial tissue-engineering applications. Previous studies have demonstrated the fabrication of vascular constructs using a collagen-based core ink and an alginate-based shell bioink for tissue-engineering applications [12]. Additionally, 3D-printed hydrogel scaffolds incorporating hyaluronic acid and gelatin have demonstrated enhanced hemostatic and antibacterial properties [13]. Chitosan-based composite scaffolds integrated with 3D-printed frameworks have been shown to mimic the structural and viscoelastic properties of native bone ECM [14]. Furthermore, gelatin-based composite wound dressings reinforced with natural biomaterials have attracted considerable attention because of their enhanced mechanical properties and biological functionality [15,16]. Despite these advantages, many natural polymer-based hydrogels suffer from limited mechanical stability. For example, collagen and gelatin generally possess insufficient mechanical strength, whereas silk fibroin, chitosan, and other natural polymers often require additional modification to improve printability and scaffold stability [17,18,19].

**Figure 1:**
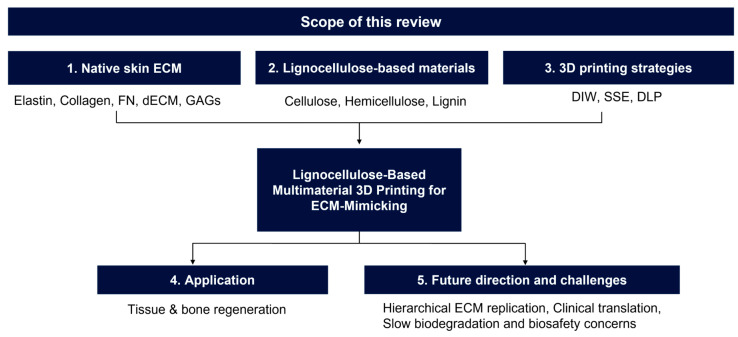
Scope of this review.

To overcome these limitations, lignocellulose-based multimaterial hydrogels have emerged as a promising approach for enhancing biomimetic properties. Lignocellulose is a biopolymeric material composed of cellulose, hemicellulose, and lignin [20]. Hemicellulose is an amorphous polysaccharide with excellent water-retention capacity. Cellulose provides excellent mechanical strength through hydrogen-bonding interactions and forms fibrous network structures, whereas lignin contributes antimicrobial activity [21,22,23]. Lignocellulosic materials have been widely employed to modify composite systems owing to their favorable mechanical properties, rich functional-group chemistry, and good biocompatibility [24,25]. Moreover, the inherently insoluble cellulose-based nanostructures in lignocellulosic materials enhance printability and gel stability at low concentrations [26]. In addition, lignocellulose-based composite hydrogels have been reported to support cell adhesion and proliferation while maintaining structural integrity during long-term culture [27]. Furthermore, compared with collagen- and alginate-based hydrogels, lignocellulosic materials can provide improved mechanical stability and slower degradation [28,29,30]. For example, single-component collagen scaffolds generally exhibit Young’s moduli of approximately 92–390 kPa under hydrated conditions, whereas lignocellulose-based scaffolds have been reported to exhibit mechanical properties ranging from several MPa to the GPa range depending on cellulose content and processing strategy [31,32]. However, the advantages of lignocellulosic materials are accompanied by several challenges. While collagen-, gelatin-, and alginate-based biomaterials typically degrade within weeks to months, depending on composition and crosslinking conditions [33,34], lignocellulosic materials exhibit slower degradation [35]. Consequently, the long-term fate and biocompatibility of degradation products require further investigation. These characteristics make lignocellulose-based materials suitable for application in 3D-printed hydrogel platforms. However, their application in mimicking ECM complexity within 3D-printed multimaterial systems remains underexplored. Existing reviews have primarily focused on lignocellulosic biomaterials, hydrogel formulations, or bioprinting technologies as separate topics [36,37]. However, the integration of lignocellulosic materials into multimaterial 3D printing strategies to recreate the structural and compositional heterogeneity of the ECM remains largely underexplored. This review links the biological functions of native ECM components with the physicochemical properties of cellulose, hemicellulose, and lignin. It further examines how multimaterial printing strategies can be used to create ECM-mimicking architectures for tissue engineering. Figure 1 presents the overall scope and framework of this review.

## 2. Literature Search Strategy

The literature discussed in this review was collected from Web of Science, Scopus, PubMed, and Google Scholar. Searches were conducted using terms related to lignocellulosic biomaterials, extracellular matrix (ECM)-mimicking scaffolds, wound healing, hydrogels, and 3D printing technologies. Representative keywords included “lignocellulose”, “cellulose nanofiber”, “cellulose nanocrystal”, “lignin”, “hemicellulose”, “ECM”, “wound healing”, “hydrogel”, and “3D bioprinting”. Both original research articles and review papers relevant to lignocellulose-based materials and ECM-mimicking scaffold fabrication were considered. Greater emphasis was placed on recent studies, while earlier publications were included when they provided important background information or foundational concepts.

## 3. Native Skin ECM and Biomimetic Design

### 3.1. Native Skin ECM Structure

**Figure 2:**
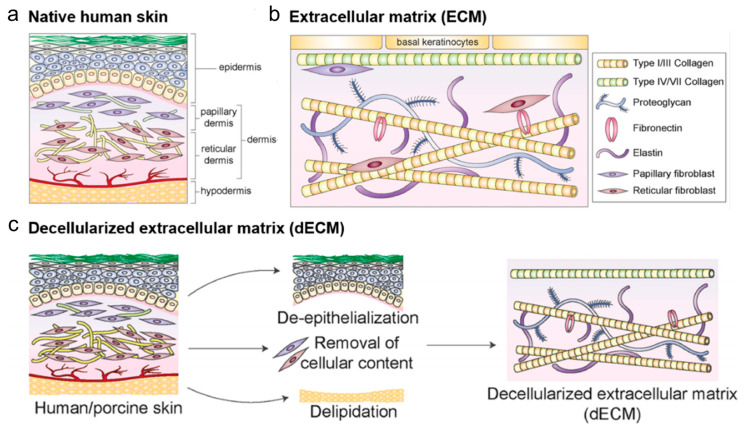
Schematic overview of (**a**) native human skin architecture, (**b**) ECM composition, and (**c**) decellularized ECM (dECM) generation as a basis for biomimetic design, reproduced with permission from ref. [50].

The dermis contains fibroblasts, vascular and lymphatic networks, nerve fibers, and a complex extracellular matrix (ECM). The ECM is a complex network composed of fibrous proteins, glycoproteins, proteoglycans, and glycosaminoglycans (GAGs), which collectively provide structural and biochemical support to surrounding cells and tissues [5]. Figure 2 illustrates the structure of native skin and the decellularization process used to obtain dECM while preserving the native extracellular matrix architecture. The ECM regulates cellular behavior through biochemical signaling, supports immune cell recruitment and migration, and plays a central role in wound healing [38]. These characteristics have motivated the use of ECM-derived biomaterials for 3D skin bioprinting. Collagen and decellularized ECM (dECM), as well as collagen-derived gelatin, are frequently employed to create skin constructs that more closely resemble native tissue [39,40,41,42]. As a result, ECM-derived materials such as collagen, gelatin, hyaluronic acid, and dECM have been widely incorporated into bioinks for the fabrication of tissue-mimetic skin constructs [36,43]. Lignocellulosic materials possess structural and functional features that resemble those of native ECM components. The correspondence between native ECM components and lignocellulosic materials is summarized in Table 1. Compared with collagen, gelatin, hyaluronic acid, alginate, and dECM, lignocellulosic materials generally provide greater mechanical stability and structural support [26]. Nevertheless, their biological activity is limited because they lack intrinsic cell-binding motifs and tissue-specific biochemical cues [44,45]. Consequently, lignocellulosic materials can improve mechanical performance but typically need to be combined with ECM-derived components to achieve comparable biological functionality [46,47,48,49]. As a result, bioinks containing ECM-derived materials, including collagen, gelatin, hyaluronic acid, and dECM, have been extensively developed to recreate the structural and biological features of native skin tissue.

#### 3.1.1. Collagen

Collagen assembles into organized, hierarchical architectures, including fibrillar structures (types I, II, and III) and network-forming structures (type IV) [61]. Collagen is the most abundant structural protein in the skin ECM and plays a central role in regulating cell adhesion, migration, and tissue remodeling during wound healing [62,63,64,65]. Moreover, collagen induces platelet activation and aggregation, leading to fibrin formation at the site of injury. These processes contribute to neovascularization and epithelial regeneration during tissue repair [1,66]. Owing to its biological and physicochemical properties, collagen has been widely used in tissue engineering as a scaffold for tissue repair and a structural matrix. Collagen-based biomaterials can partially reproduce the fibrillar architecture of native dermal ECM [67]. However, collagen hydrogels often exhibit limited mechanical stability and rapid degradation, necessitating their combination with reinforcing biomaterials [68]. Collagen-based wound dressings and hydrogels are often combined with natural and synthetic polymers, such as hyaluronic acid (HA), chitosan, and alginate [65,69,70,71]. These composite systems improve structural durability while preserving the ability of collagen to modulate inflammatory responses, support ECM remodeling, and promote new ECM formation during wound healing [72]. This strategy addresses one of the major limitations of collagen-based hydrogels, namely their limited mechanical stability under physiological conditions.

#### 3.1.2. Elastin

Elastin is a durable protein that forms the amorphous core of elastic fibers in the dermis and exhibits hydrophobic properties, providing resistance to acidic and alkaline environments [73,74,75]. After enzymatic crosslinking of tropoelastin, elastin forms resilient elastic fibers that preserve tissue elasticity under repeated deformation [76]. This mechanical function is particularly important during wound healing, where elastin contributes to dermal regeneration and helps limit excessive wound contraction [77,78,79]. The unique elastic properties of elastin have led to the development of biomaterials that replicate its elasticity and durability, particularly in hydrogel and scaffold systems for tissue regeneration [80,81]. For example, elastin-like polypeptide (ELP)-based bioinks functionalized with cell-adhesive peptides or collagen have shown favorable printability and mechanical performance [82]. They also support cell viability, highlighting the applicability of elastin-derived materials in ECM-mimetic scaffold design. Sánchez-Cid et al. demonstrated that incorporating elastin into PCL scaffolds enhanced wettability, cellular viability, and mechanical properties, supporting elastin’s potential as a functional component [83].

#### 3.1.3. GAGs

GAGs are a class of linear polysaccharides found in the extracellular matrix that play key roles in maintaining tissue hydration and mediating biological signaling. Their high density of negative charges enables water retention and facilitates interactions with proteoglycans and other biomolecules [84,85]. As a result, GAG-based biomaterials have been widely investigated for tissue-engineering applications, particularly in cartilage, bone, and wound-healing systems [86,87,88]. In particular, HA accounts for a large proportion of GAGs in the skin and plays an important role in maintaining moisture and elasticity [89]. HA is widely used in wound dressings and hydrogel systems because it supports a moist microenvironment and helps regulate inflammatory responses [90,91]. In addition, HA promotes cell migration, proliferation, and angiogenesis, making it a valuable component for ECM-mimetic biomaterials [92,93]. The chemical structure of HA provides multiple reactive sites for functionalization, enabling the modulation of its physicochemical and biological characteristics [94]. Moreover, HA is frequently incorporated into composite systems with natural or synthetic polymers to enhance scaffold integrity and biofabrication performance while preserving biocompatibility [95]. In addition, HA has been widely incorporated into lignocellulose-based multimaterial 3D printing systems because of its favorable biological properties [96].

#### 3.1.4. Fibronectin (FN)

FN is a major extracellular matrix protein that forms a fibrillar network and plays a central role in extracellular matrix maturation, cell adhesion, and cell migration during wound healing [97,98]. Following tissue injury, fibronectin contributes to the formation of a provisional matrix that provides early structural support and guides cellular infiltration into the wound site [99,100]. Fibronectin contains key cell-binding domains, notably the Arg–Gly–Asp (RGD) and the Pro–His–Ser–Arg–Asn (PHSRN) sequences, which play essential roles in mediating cell adhesion [101]. RGD and PHSRN motifs promote cell adhesion and tissue regeneration, making them attractive candidates for biomaterial functionalization [98,102]. These peptide motifs have been widely incorporated into bioinks and scaffold materials to improve cell–material interactions [103,104].

#### 3.1.5. dECM

Decellularization removes cellular components while preserving the extracellular matrix framework [105]. Removal of these cellular elements reduces immune responses while preserving the structural framework and biochemical cues of the ECM [106], thereby retaining the native architecture and tissue-specific characteristics of the original matrix [107,108]. For example, skin-derived dECM retains matrix proteins and growth factors associated with cutaneous tissue, whereas bone-derived dECM preserves osteogenic components. As a result, dECM provides a biologically relevant microenvironment that more closely resembles native tissues than many conventional biomaterials. Owing to these features, dECM-based biomaterials have been increasingly investigated as promising candidates for regenerative therapies [109,110]. dECM scaffolds are often combined with natural or synthetic polymers to improve mechanical strength for tissue-engineering applications [111]. Furthermore, functionalization of dECM-based scaffolds with growth factors and nanoparticles has been reported to enhance their regenerative capacity by stimulating key processes involved in tissue repair, including angiogenesis, cell proliferation, and matrix remodeling [112,113,114]. Despite these advantages, the broader application of dECM-based scaffolds remains constrained by batch-to-batch variability [115]. These limitations complicate standardization and may hinder large-scale clinical translation.

## 4. Lignocellulose-Based Materials for Biomedical Use

Lignocellulose is a plant biomass available in large quantities and is a composite polymer material with cell walls composed of cellulose, hemicellulose, and lignin [116]. Lignocellulose has long been utilized in a wide range of applications, including energy production and the manufacture of cellulose fibers [24,117,118]. However, its relatively low solubility limits its biomedical applications. These constraints can be mitigated through physical and chemical modifications, thereby expanding its scope of application [119,120]. In addition, functionalization with RGD and PHSRN motifs enhances cell adhesion and improves the biomimetic properties of lignocellulose-based scaffolds [121,122,123]. Such modifications could compensate for the limited intrinsic bioactivity of lignocellulosic materials and promote more effective cell–material interactions.

### 4.1. Lignocellulose Component

#### 4.1.1. Cellulose

**Figure 3:**
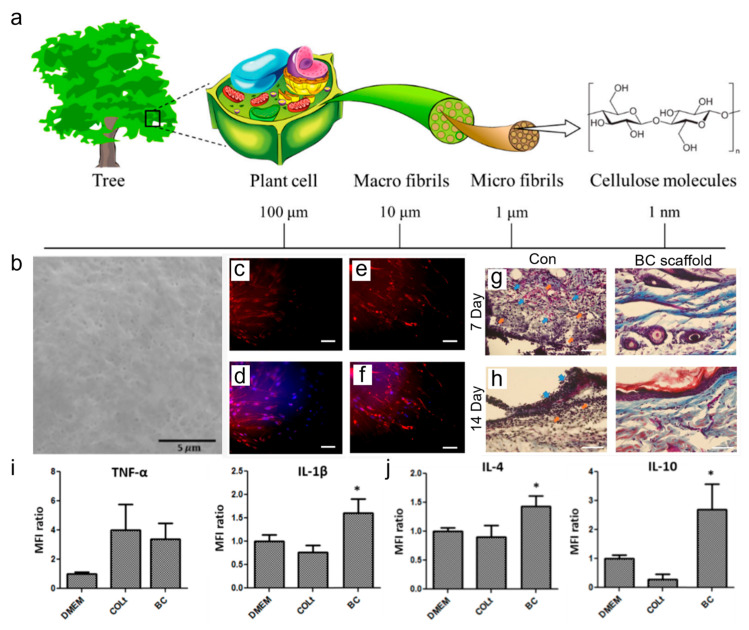
(**a**) Hierarchical structure of cellulose [125]. Arrows indicate the hierarchical structure of cellulose, and dashed lines represent magnified views of each structural level. (**b**) SEM image of a BC scaffold. Immunofluorescence staining of human adipose-derived stem cells (hASCs) cultured on BC scaffolds for 14 days, showing (**c**) OCT-4 expression, (**d**) OCT-4/Hoechst overlay, (**e**) Nestin expression, and (**f**) Nestin/Hoechst overlay. Histological evaluation of rat skin defects using Masson’s trichrome staining after (**g**) 7 and (**h**) 14 days of treatment. Quantitative analysis of inflammatory cytokine expression, including (**i**) M1-associated markers and (**j**) M2-associated markers, highlighting the ability of BC scaffolds to support stem cell activity and regulate the wound-healing microenvironment [135] (* *p* ≤ 0.05).

Cellulose is the most abundant natural biopolymer and, owing to its hierarchical and multidimensional architecture, supports a wide range of functional properties and application potentials [124]. Figure 3a illustrates the hierarchical structure of cellulose, spanning from plant tissue to plant cells, macrofibrils, microfibrils, and ultimately individual cellulose molecular chains [125]. The distinctive molecular architecture of cellulose provides high mechanical strength and water affinity, facilitating exudate absorption and tissue repair [126,127]. Cellulose is also used in various forms, including cellulose nanocrystals (CNCs), cellulose nanofibers (CNFs), and bacterial cellulose (BC). CNCs are characterized by a highly ordered crystalline structure, superior mechanical strength, and a large specific surface area [128]. CNF is a nanocellulose material composed of interconnected fibrillar networks and exhibits an elastic modulus exceeding 114 GPa, making it suitable for reinforcing composite materials [129,130]. As collagen-based scaffolds are widely used in tissue engineering but often lack sufficient mechanical strength for long-term cell culture [31], the higher elastic modulus of CNF highlights its potential for improving scaffold mechanical stability. BC is biosynthesized extracellularly by bacteria, eliminating the need for complex extraction processes [131]. Cellulose-based hydrogels can significantly enhance the mechanical properties of ECM-inspired scaffolds while maintaining biocompatibility and biodegradability, leading to their widespread use in skin, bone, and cartilage tissue engineering [132,133,134]. A previous study developed a bacterial cellulose scaffold with a nanofibrous architecture resembling native ECM [135]. The BC scaffold exhibited a smooth and interconnected surface morphology (Figure 3b). Expression of OCT-4 and Nestin indicated that the BC scaffold supported stem cell maintenance and differentiation (Figure 3c–f). The ECM-like nanofibrous architecture of bacterial cellulose likely contributed to these cellular responses by promoting cell adhesion and cell–material interactions [136]. These findings suggest that the nanofibrous architecture of BC not only provides structural support but also promotes the establishment of a microenvironment conducive to cell adhesion, proliferation, and tissue regeneration. In vivo studies demonstrated that BC-scaffold-treated wounds exhibited enhanced healing after 7 days, as evidenced by increased keratinocyte formation and reduced inflammatory cell infiltration (Figure 3g,h). Furthermore, analysis of macrophage markers indicated that the BC scaffold influenced both M1 and M2 macrophage polarization, highlighting its potential to modulate the wound-healing microenvironment (Figure 3i,j). These findings suggest that the therapeutic benefits of BC are not solely attributable to its mechanical support. Rather, its nanofibrous architecture promotes cell–material interactions and modulates macrophage behavior, contributing to wound-healing regulation.

#### 4.1.2. Hemicellulose

**Figure 4:**
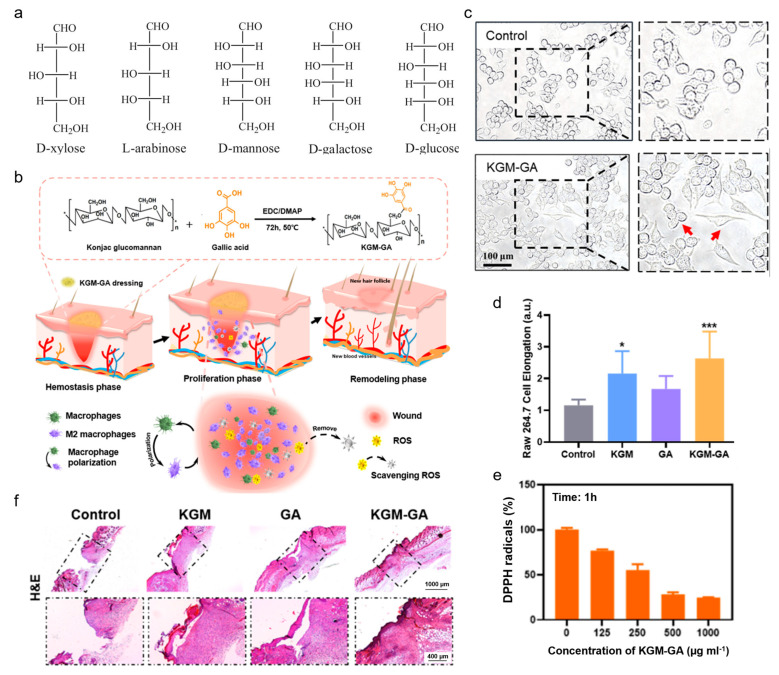
(**a**) Monosaccharides constituting the hemicellulose structure [138]. (**b**) Schematic illustration of KGM-GA synthesis and its role in wound healing via M2 polarization and ROS scavenging. (**c**) Morphology of macrophage after 48 h exposure, showing M2-like polarization. Red arrowheads denote macrophages exhibiting round and elongated morphologies. (**d**) Quantification of macrophage elongation associated with M2-like polarization. Mean ± SD, *n* = 10; * *p* < 0.05, *** *p* < 0.001 vs. control. (**e**) Free radical scavenging activity of KGM, GA, and KGM–GA. (**f**) H&E staining of wound tissues at day 7, reproduced with permission from ref. [143].

Hemicellulose is one of the most abundant biopolymers in nature, second only to cellulose. Hemicellulose is composed of a structurally diverse set of monosaccharides and uronic acids, such as D-xylose, D-mannose, and L-arabinose, among others [137] (Figure 4a). Hemicellulose contains abundant hydroxyl groups, resulting in high hydrophilicity that can limit its practical applications [138]. Therefore, chemical modification and reinforcement with other biomaterials are required to improve polymer compatibility, scaffold stability, and the overall applicability of hemicellulose-based biomaterials [57,138,139,140,141]. Hemicellulose-based nanoaggregates have been incorporated into dual-network composite hydrogels to improve mechanical performance, stimulus responsiveness, and sustained drug release [142]. These results highlight the potential of hemicellulose to enhance mechanical performance and enable controlled drug delivery. However, additional bioactive components may be required to achieve more active regulation of the wound-healing process. Li et al. developed a hemicellulose-based KGM–GA hydrogel, composed of konjac glucomannan (KGM) and gallic acid (GA), to regulate the wound microenvironment [143] (Figure 4b). The KGM–GA hydrogel promoted macrophage polarization toward an M2-like phenotype compared with the control group (Figure 4c,d), suggesting its potential to establish a pro-regenerative microenvironment [144]. In addition, KGM–GA also exhibited ROS-scavenging activity (Figure 4e), which may help alleviate oxidative stress associated with delayed wound healing [145]. Consistent with these effects, the KGM–GA group showed enhanced re-epithelialization and granulation tissue formation compared with the control group (Figure 4f). These improvements are likely attributable to the anti-inflammatory and antioxidant activities of KGM–GA rather than its role as a structural scaffold alone.

#### 4.1.3. Lignin

**Figure 5:**
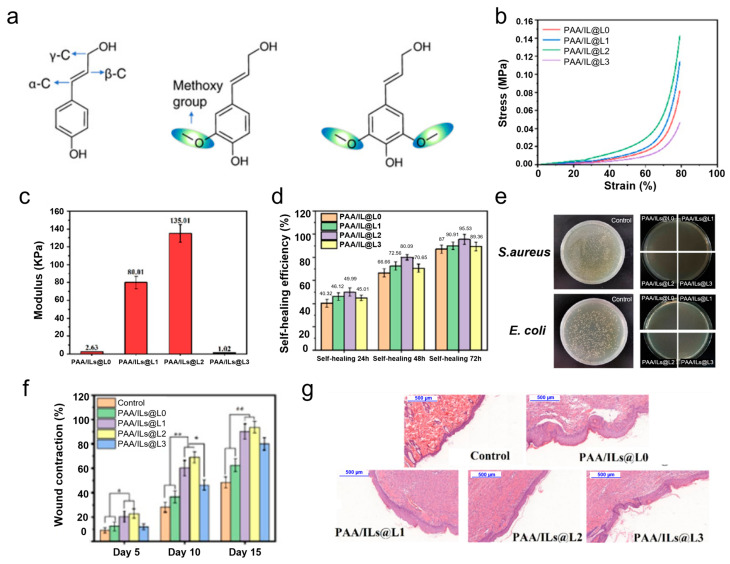
(**a**) Structure of lignin [146]. (**b**) Compressive stress–strain curves and (**c**) Young’s modulus of hydrogels demonstrating the reinforcing role of lignin. (**d**) Quantitative assessment of self-healing efficiency. Statistical significance was calculated by one-way ANOVA using the Tukey post-hoc (* *p* ≤ 0.05, ** *p* ≤ 0.01). (**e**) Representative bacterial colony images. (**f**) Wound closure was evaluated by contraction area on days 5, 10, and 15. (**g**) Histological staining images of the hydrogel-treated groups on day 15, reproduced with permission from ref. [154].

Lignin is a heterogeneous polymer characterized by a naturally branched architecture, and it is primarily composed of three fundamental monolignol units: syringyl (S), guaiacyl (G), and p-hydroxyphenyl (H) [146] (Figure 5a). Lignin has attracted attention as a functional component for hydrogel-based biomaterials because of its antioxidant activity, biocompatibility, and strong UV-absorbing properties [147,148]. Lignin requires separate extraction and purification processes for industrial utilization. Various extraction and processing methods have enabled the conversion of lignin into functional forms suitable for biomedical applications [149]. Lignin obtained through this process can be developed into various forms, such as nanoparticles, nanofibers, and hydrogels, and applied to a wide range of industrial fields [150,151,152]. A lignin-based hydrogel with controlled, sustained-release capabilities and enhanced antioxidant and antibacterial activities has been reported [153]. A study reported a sulfonated lignin-based supramolecular hydrogel (PAA/ILs@L2) dressing that demonstrated enhanced self-healing capability and bioactivity, with potential for wound healing [154]. PAA/ILs@L2 maintained its structural integrity under compressive deformation and exhibited improved mechanical properties compared with the control formulations (Figure 5b,c), indicating that lignin effectively reinforced the hydrogel network [155]. Furthermore, PAA/ILs@L2 showed efficient self-healing behavior and antibacterial activity (Figure 5d,e). In vivo studies further demonstrated enhanced wound healing and tissue regeneration, including improved re-epithelialization and skin appendage formation (Figure 5f,g). These findings highlight the potential of lignin to provide both structural reinforcement and bioactive functions in wound-healing biomaterials. These properties have supported the growing use of lignin in hydrogel-based biomaterials and wound-healing applications. However, variability arising from different extraction methods remains a challenge.

## 5. 3D Printing Strategies for Lignocellulose-Based Multimaterial Composites

3D printing is widely utilized to fabricate hydrogel-based constructs with well-defined and tunable architectures. By adjusting key parameters such as pore size, filament spacing, and crosslinking density, it is possible to regulate cellular behaviors, including adhesion, proliferation, and migration [156]. These design capabilities enable the creation of porous, grid-like, or fiber-mimicking network structures that resemble the native ECM microenvironment [157,158,159]. Multimaterial 3D printing enables the integration of two or more distinct materials within a single construct, allowing the formation of architectures with spatially varied compositions [160]. Gradient scaffolds mimic the compositional and structural transitions found in native tissues through spatial variations in material properties. These designs can improve tissue integration and regeneration by more closely replicating the hierarchical organization of biological tissues [161]. For example, Zhang et al. developed a multileveled gradient hydrogel for osteochondral repair by incorporating superparamagnetic hydroxyapatite nanorods into an adaptable GelMA/AcCD double-network hydrogel [162]. The scaffold generated continuous gradients in mineral content and mechanical properties, thereby mimicking the transition from cartilage to subchondral bone. A hydroxyapatite (HAp)-gradient PVA/bacterial cellulose scaffold was developed using a buoyancy-driven gradient method to mimic the mineral gradient of native bone tissue [163]. The gradient distribution of HAp enhanced cell adhesion, proliferation, osteogenic differentiation, and biocompatibility compared with homogeneous scaffolds, highlighting the potential of lignocellulose-based gradient architectures to recapitulate tissue heterogeneity and support site-specific tissue regeneration. Beyond static gradient designs, adaptive scaffolds can dynamically respond to changes in the local microenvironment and provide stage-specific biological cues during tissue repair [164]. For instance, self-adaptive nanotopographies that dynamically promote biomimetic mineral deposition have been reported to generate ECM-like microenvironments that enhance osteogenesis and tissue regeneration [165]. Adaptive lignocellulose-based scaffolds have recently been developed by combining nanocellulose with stimuli-responsive polymers [166]. These scaffolds responded to changes in pH, temperature, and ionic conditions, demonstrating their potential for dynamic regulation of the cellular microenvironment.

However, processing multiple materials increases fabrication complexity and may compromise interfacial compatibility and structural stability [167]. In particular, the heterogeneous composition of lignocellulose can complicate its integration with synthetic polymers, resulting in reduced compatibility and weak interfacial adhesion [168,169]. To mitigate these limitations, various chemical modification strategies and gradient printing approaches have therefore been employed to improve material integration and reduce interfacial mismatches [170,171,172]. These challenges highlight the importance of selecting appropriate printing techniques for lignocellulose-based constructs. Table 2 provides an overview of ECM-mimetic hydrogels fabricated using various 3D printing techniques. Although fused deposition modeling (FDM), direct ink writing (DIW), semi-solid extrusion (SSE), stereolithography (SLA), and digital light processing (DLP) have all been used to fabricate lignocellulose-based scaffolds, each technique has distinct strengths and limitations. Among these techniques, FDM is advantageous for producing mechanically robust constructs and large-scale fabrication, whereas DIW and SSE are better suited for hydrogel-based bioinks and cell-compatible processing [173,174,175,176]. SLA and DLP provide higher resolution and shape fidelity, although their broader application remains limited by the availability of photocurable lignocellulose-based formulations and concerns regarding photoinitiator biocompatibility [177,178,179,180]. Furthermore, process standardization, manufacturing reproducibility, and regulatory approval remain major challenges for clinical translation [181].

### 5.1. Extrusion-Based 3D Printing Techniques

In extrusion-based bioprinting, the rheological conditions of bioinks are highly demanding [189]. Appropriate viscosity is required to maintain filament continuity and printing fidelity, whereas excessively high viscosity can increase shear stress during extrusion, potentially leading to nozzle clogging [190]. To ensure stable printing and shape retention, bioinks are generally formulated with appropriate viscoelastic properties, such as shear-thinning behavior and adequate yield stress [191]. Printing performance is further influenced by processing parameters, including temperature, nozzle diameter, extrusion pressure, and printing speed, which collectively determine filament formation and structural fidelity [192,193,194]. Crosslinking also plays an important role in maintaining the geometry of printed constructs after fabrication [195]. In addition, post-printing stability and degradation behavior should be considered to preserve scaffold integrity while allowing cell growth and tissue remodeling [196,197]. Therefore, precise control of these parameters is critical for reproducing ECM-like microarchitectures and maintaining the structural integrity of multilayer constructs. The application of FDM to ECM-mimicking lignocellulose scaffold fabrication remains limited because the technique primarily relies on thermoplastic materials, which are often incompatible with cell-laden and hydrogel-based bioinks [198]. Therefore, this section focuses primarily on DIW and SSE printing strategies.

#### 5.1.1. DIW

DIW fabricates structures by extruding materials through a nozzle under controlled pressure and has been widely adopted because of its operational simplicity, cost-effectiveness, and compatibility with multimaterial fabrication [175,199]. DIW bioinks exhibit viscosities commonly on the order of 10^2^–10^6^ mPa·s at low shear rates, although the optimal range varies with material composition and printing conditions [200,201]. Depending on the material system and processing conditions, DIW can generate structures with resolutions ranging from hundreds of micrometers to the sub-millimeter scale [175]. DIW is particularly attractive for multimaterial fabrication because different bioinks can be deposited at predefined locations using multiple print heads. Radeke et al. developed transparent cellulose nanofiber (cNFC) bioinks for extrusion-based 3D printing by partially carboxymethylating cellulose fibers [202]. When combined with gelatin, the aligned cNFC fibers guided myotube orientation along the printing direction, enabling the fabrication of anisotropic muscle tissues that mimic the structural organization of native ECM. DIW has also been employed to generate bone-mimetic architectures. Li et al. developed ECM-mimicking scaffolds for bone tissue engineering by fabricating BG/cellulose composite structures with a controlled porous architecture and biocompatibility [203]. BG/cellulose composite structures exhibited an interconnected porous architecture and a rough surface morphology that mimicked the hierarchical features of native bone ECM, thereby promoting hydroxyapatite formation and cell adhesion and proliferation. DIW-based cellulose scaffolds capable of supporting ECM deposition and angiogenic activity have also been reported [204]. The printed constructs supported long-term fibroblast phenotype maintenance and enhanced the deposition of ECM proteins. Sustained secretion of angiogenic factors further highlighted their potential for recreating ECM-like microenvironments. Collectively, these studies demonstrate that DIW enables the fabrication of lignocellulose-based scaffolds with controlled architecture, anisotropic organization, and bioactive microenvironments that support tissue regeneration.

#### 5.1.2. SSE

SSE is an extrusion-based additive manufacturing technique in which gel- or paste-like materials are deposited in a layer-by-layer manner to build three-dimensional structures [205]. In contrast to FDM, SSE operates at relatively low processing temperatures, making it well-suited for fabricating constructs that incorporate living cells [206] and for combining polymers with therapeutic agents [207]. Juan et al. developed multifunctional PCL-based wound dressings incorporating curcumin, lignin, and D-panthenol using SSE 3D printing [208]. The constructs exhibited sustained drug release, antioxidant and antibacterial activities, and a porous architecture conducive to cell growth, which collectively contributed to enhanced wound healing in vivo. The ability of cellulose-based hydrogels to support long-term cell activity has also been investigated [204]. The system demonstrated that material composition and crosslinking conditions can regulate fibroblast phenotype, ECM deposition, and angiogenic growth factor secretion during long-term culture. More complex ECM-inspired architectures have also been achieved by combining hydrogel and fibrous components [209]. The scaffold provided antibacterial activity, high water absorption capacity, and good fibroblast compatibility while maintaining a moist environment favorable for wound healing. These examples highlight the potential of lignocellulose-based materials to support the development of scaffolds that more closely mimic the structural and functional characteristics of native ECM.

### 5.2. Vat Photopolymerization

SLA and DLP utilize photopolymerization to selectively solidify photosensitive resins through controlled light exposure [210]. SLA uses a focused laser to cure photocurable resin, enabling precise patterning while maintaining high printing resolution [211,212]. Although SLA can fabricate photocurable hydrogel structures, light scattering and limited resin compatibility restrict its broader application [213]. In contrast, DLP enables more uniform and efficient layer-wise curing, making it a more suitable approach for constructing lignocellulose-derived hydrogel scaffolds [214]; therefore, this section focuses primarily on DLP-based strategies.

#### DLP

**Figure 6:**
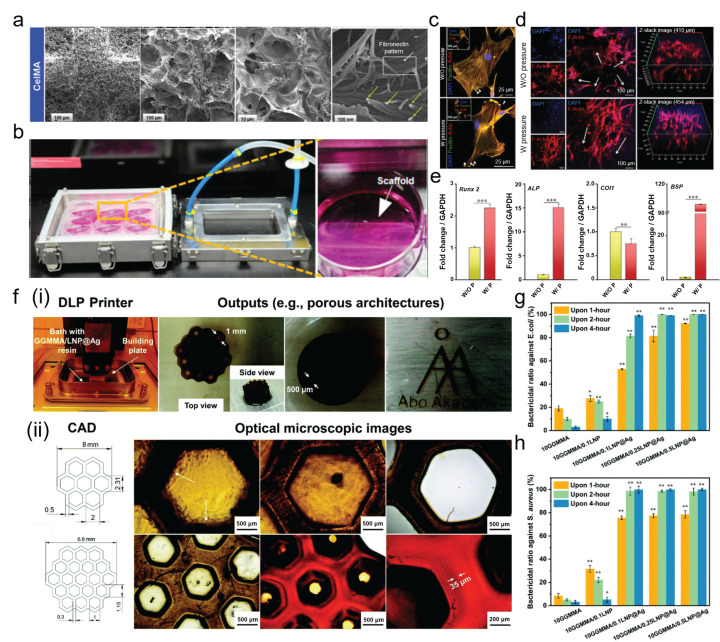
(**a**) FE-SEM images of the CelMA scaffolds showing interconnected porous architectures. The arrows indicate the fibronectin pattern. (**b**) Pulsatile pressure bioreactor configuration incorporating a cartridge reactor fitted with a six-well plate. (**c**,**d**) Assessment of the osteogenic differentiation of hBMSCs cultured with CelMA under PPS conditions in vitro. (**e**) Real-time qPCR analysis of bone formation marker genes, reproduced with permission from ref. [218]. (**f**) Reproducibility of DLP-printed structures using 10GGMMA/0.1LNP@Ag resin fabricated with an M-One Pro 30 DLP printer (Makex Co., Ltd., China): (**i**) shows honeycomb, cross-hatched, and ultrathin architectures; (**ii**) compares honeycomb scaffolds fabricated from varying compositions of resins. Analysis of the viability of GGMMA-based hydrogel against (**g**) *E. coli*, (**h**) *S. aureus* [219]. Statistical significance was calculated by one-way ANOVA using the Tukey post-hoc (* *p* ≤ 0.05, ** *p* ≤ 0.01, and *** *p* ≤ 0.001).

DLP is a 3D printing technique that solidifies liquid resin through projected light patterns [215]. DLP enables the fabrication of hydrogel structures with high resolution and accurate geometric control. Silva et al. developed a fully cellulosic photocurable resin for DLP printing and produced hydrogels that maintained their shape while exhibiting favorable mechanical properties [180]. Multimaterial fabrication in DLP printing is achieved by exchanging the liquid resin environment, typically through either tank-swapping or resin-exchange approaches [216]. These approaches enable the integration of materials with distinct properties within a single construct. For example, Chand et al. developed a biomimetic corneal stroma equivalent via DLP bioprinting with a dual-crosslinked GelMA/OxiCMC interpenetrating-network hydrogel using a LumenX+ DLP printer (CELLINK, Gothenburg, Sweden) [217]. The printed constructs reproduced the curved geometry of the native cornea and exhibited interconnected porous structures. The hydrogels also showed high optical transparency and supported keratocyte viability and proliferation. Ganguly et al. developed ECM-inspired GelMA/CNCs hydrogel scaffolds (CelMA) that were fabricated via DLP 3D printing, enabling the regulation of stem cell behavior and promoting osteogenic differentiation under a controlled pulsatile pressure stimulation (PPS) [218]. CelMA scaffolds containing CNCs exhibited porous architectures that supported cell infiltration and growth (Figure 6a). A PPS system was applied to the cell-laden hydrogels to provide controlled mechanical stimulation (Figure 6b). Under these conditions, the CelMA scaffolds enhanced osteogenic differentiation, demonstrating their potential for bone tissue engineering (Figure 6c–e). The combination of CNC-containing scaffolds and pulsatile mechanical stimulation appeared to contribute to the enhanced osteogenic response, indicating that both scaffold composition and biomechanical cues influence cell behavior. Wang et al. employed DLP-based photopolymerization to fabricate lignocellulose-derived hydrogels (GGMMA/LNP@Ag) that exhibit ECM-mimicking characteristics through their hydrated network and porous architecture [219]. The resin was fabricated into well-defined porous architectures, including honeycomb and lattice structures (Figure 6f), which mimic key structural features of native ECM. Furthermore, controlled silver-ion release from the lignin-based nanocomposite provided antibacterial activity, highlighting its potential for wound-healing applications (Figure 6g,h). The results indicate that lignocellulos-based nanocomposites can support scaffold fabrication while simultaneously providing antibacterial functionality, which may be advantageous for wound-healing applications.

## 6. Long-Term Bioactivity of Lignocellulose Materials

### 6.1. Cell–Material Interaction

**Figure 7:**
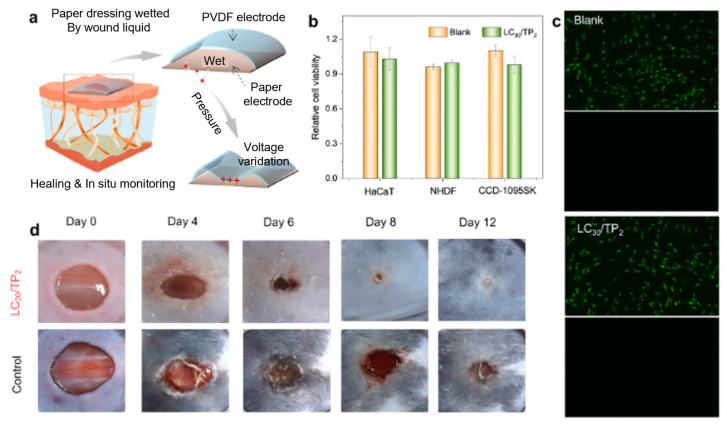
(**a**) Overview of the lignocellulose paper dressing system developed for wound management and healing monitoring. The “+” symbols denote positive charges, and the arrows indicate the direction of pressure application. (**b**) Cell viability results obtained from various cell lines after treatment with different dressing formulations. (**c**) Live/dead staining images demonstrating cellular responses to the LC30/TP2 dressing compared with the control group. (**d**) Representative photographs illustrating wound healing progression in an excisional wound model [223].

The interaction between lignocellulosic biomaterials and surrounding cells plays a critical role in determining their long-term bioactivity and regenerative performance [220]. Cell–material interactions influence cell adhesion, migration, proliferation, angiogenesis, and ECM remodeling, which collectively contribute to tissue regeneration and wound healing [221]. 3D-printed functionalized cellulose scaffolds enhanced stem cell migration, angiogenesis, and re-epithelialization, which are critical processes underlying effective wound repair in diabetic wound models [222]. Similar findings have been reported for other lignocellulose-derived systems. For example, a lignocellulose-based paper dressing containing tea polyphenols (TPs) exhibited excellent cytocompatibility toward human epidermal keratinocytes (HaCaT), normal human dermal fibroblasts (NHDF), and CCD-1095SK cells [223] (Figure 7a–c). In vivo studies additionally demonstrated that favorable cell–material interactions contributed to enhanced tissue regeneration (Figure 7d). Likewise, a 3D-printed carboxymethyl cellulose (CMC)/ε-polylysine hydrogel maintained fibroblast viability above 98% and enhanced cell proliferation during culture [224]. In vivo studies further demonstrated that the hydrogel promoted granulation tissue formation, collagen deposition, angiogenesis, and ECM remodeling, thereby enhancing overall wound healing. These observations suggest that lignocellulosic materials can provide a favorable microenvironment for cellular activities and tissue reconstruction. Notably, reduced inflammatory cell infiltration, increased vascularization, and enhanced collagen deposition have been repeatedly observed across different lignocellulose-based platforms [224,225]. Such responses may contribute to the transition from the inflammatory phase to the proliferative and remodeling phases of wound healing. Despite these promising findings, current evidence is largely derived from short-term in vitro studies and small-animal wound models. Consequently, it remains difficult to determine whether the observed cellular responses can be maintained during long-term implantation or translated into clinically relevant outcomes. Future research should aim to clarify the molecular basis of cell–material interactions while establishing standardized evaluation strategies for long-term tissue integration, biological safety, and regenerative performance.

### 6.2. Biodegradation

Biodegradation is a critical factor in the long-term performance of biomaterials. Excessively rapid degradation may compromise mechanical integrity, whereas slow degradation can result in long-term material retention and unpredictable biological responses. Hemicellulose is typically more susceptible to biodegradation owing to its amorphous structure [139,226]. Cellulose is also biodegradable. However, its partially crystalline organization generally results in a slower degradation rate than that of hemicellulose [227]. In contrast, the aromatic structure of lignin makes it more resistant to degradation than other lignocellulosic components [228]. Although this may improve scaffold stability, it also complicates the prediction of long-term degradation behavior and biological fate after implantation. Several studies have demonstrated the favorable biocompatibility of lignin-containing biomaterials in vivo, with no obvious signs of toxicity [229,230]. However, these studies primarily focus on short- to medium-term implantation and tissue regeneration outcomes, and information regarding the fate of lignin-derived degradation products and their long-term biological effects remains limited. Therefore, further studies are needed to clarify the degradation mechanisms of lignin-based biomaterials, identify their degradation products, and evaluate their biodistribution and clearance pathways. Furthermore, direct quantitative comparison of degradation behavior remains challenging because of differences in material composition and evaluation protocols. Standardized assessment methods are needed to establish reliable degradation benchmarks. Such investigations will be important for assessing the long-term safety and clinical applicability of lignin-containing biomaterials.

### 6.3. Immunogenicity

**Figure 8:**
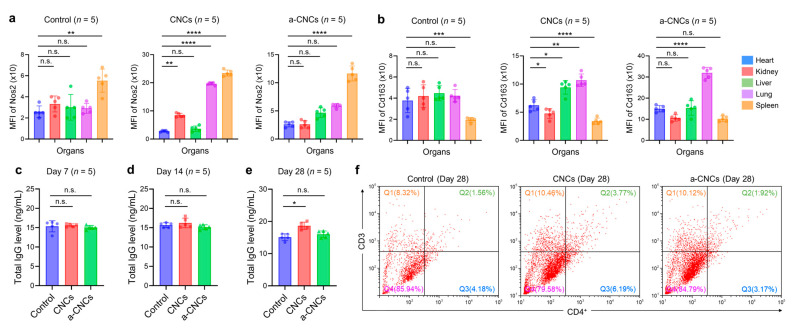
(**a,b**) MFI-based quantification of Nos2 and Cd163 expression in major organs (*n* = 5). (**c**–**e**) Serum IgG levels monitored over 28 days, indicating the absence of significant adaptive immune activation (*n* = 5). (**f**) Quantification of CD4^+^ T-cell populations 28 days after administration of PBS, pristine CNCs, or a-CNCs, showing no substantial treatment-induced immune activation (*n* = 5) [233]. Statistical significance was calculated by one-way ANOVA using the Tukey post-hoc (* *p* ≤ 0.05, ** *p* ≤ 0.01, *** *p* ≤ 0.001, and **** *p* ≤ 0.0001).

The immunogenicity of biomaterials is an important factor influencing their long-term safety and clinical performance [231]. Current evidence suggests that cellulose-based nanomaterials exhibit relatively low acute immunotoxicity. While the long-term retention of CNFs in lung tissue and their potential to induce mild inflammatory responses warrant further investigation, most studies have reported limited cytotoxicity toward macrophages and minimal systemic immune activation [232]. Amide-functionalized CNCs (a-CNCs) showed no detectable systemic inflammatory or adaptive immune responses in vivo and promoted M2 macrophage polarization, suggesting favorable immunocompatibility [233]. Notably, macrophage viability remained above 95%, while CD206 and IL-10 expression increased by more than 15-fold without significant changes in serum IgG levels or CD4^+^/CD8^+^ T-cell ratios (Figure 8a–f). Similarly, the immune response to lignin-based materials appears to be highly dependent on their physicochemical properties and surface functionalization. For instance, peptide-functionalized lignin nanoparticles were designed to target M2-like macrophages and reprogram them toward a pro-inflammatory M1 phenotype, thereby enhancing antitumor immune responses [234]. Furthermore, purified lignin and lignin nanoparticles generally exhibit low cytotoxicity toward macrophages and minimal hemolytic activity, indicating a low risk of acute immune-related adverse effects [235,236]. Taken together, current studies suggest that lignocellulosic biomaterials generally exhibit favorable immunocompatibility while retaining the capacity to regulate macrophage functions. Nevertheless, differences in particle size, surface chemistry, dosage, and administration route make direct comparisons among studies difficult. Moreover, despite the generally low acute immunogenicity reported to date, uncertainties regarding long-term accumulation, degradation products, and chronic immune responses remain. Therefore, comprehensive biosafety assessments will be essential for future clinical translation.

## 7. Future Directions and Challenges

3D printing has been widely investigated as a platform for recreating ECM-mimetic architectures that support wound healing while enabling the fabrication of patient-specific constructs. Lignocellulosic materials have attracted increasing interest because of their renewability and broad potential for chemical modification. Nevertheless, several challenges continue to limit their broader application in 3D bioprinting.

First, the lack of evaluation methods to quantitatively assess ink printability limits direct comparisons between studies. Therefore, it is difficult to identify a clear correlation between ink composition and printing accuracy and to determine the impact on biological performance. Such limitations are more pronounced in lignin-based materials, as their complex structure leads to reduced reproducibility. Additionally, the limited range of lignin-based materials applicable to photopolymerization-based processes (SLA/DLP) is cited as a major technical limitation. Second, current 3D printing technology still has limitations in precisely reproducing the complex hierarchical structures and mechanical properties found in biological tissues. Multimaterial printing enables the fabrication of complex tissue-like architectures. However, differences in bioink viscosity and rheological properties can cause nozzle clogging and cross-contamination, ultimately reducing printing accuracy and reproducibility. Third, the slow biodegradation rate of cellulose-based structures may increase the risk of chronic inflammation or foreign body reactions during long-term implantation. Although promising results have been reported, differences in material composition, modification strategies, and evaluation methods make it difficult to compare degradation behavior and immunogenicity across studies. Consequently, the long-term safety of lignocellulose-based scaffolds remains insufficiently understood.

The integration of lignocellulosic materials with smart hydrogels has expanded the design of wound-responsive scaffolds. Stimulus-responsive lignocellulosic biomaterials capable of controlled drug release, wound monitoring, and microenvironment regulation have been explored for advanced wound care applications [223,237]. These systems may enable printed scaffolds to better adapt to changes in the wound environment. However, challenges related to long-term functional stability, scalable manufacturing, sterilization compatibility, and regulatory approval remain unresolved. Addressing these issues will be important for the clinical application of adaptive lignocellulose-based scaffolds.

## Figures and Tables

**Table 1:** Native ECM functions and corresponding lignocellulosic biomimetic features.

Native ECM Component	Function in ECM	Lignocellulosic Component	Potential Contribution to ECM-Mimetic	Refs.
Collagen	Structural support and mechanical strength	Cellulose	Structural integrity and load-bearing capacity	[51,52,53,54]
Elastin	Elasticity and resilience	Cellulose composites	Improves flexibility and mechanical recovery	[55,56]
GAGs	Hydration and molecular transport	Hemicellulose	Enhances water retention and swelling behavior	[57,58]
Fibronectin	Promotes cell adhesion, migration, and proliferation	Functionalized cellulose	Provides adhesive interfaces and enhances interactions with surrounding tissues	[59,60]

**Table 2:** Summary of representative 3D printing strategies for ECM-mimetic hydrogel fabrication.

Printing Method	Crosslinking Mechanism	ECM-Mimicking Feature	Application	Functional Mechanism	Limits	Ref.
DIW	LOX-mediated enzymatic	Porous structure and mineral formation	Diabetic bone regeneration	Promoting the bone regeneration microenvironment through the regulation of intrafiber mineralization arrangement	Limited evaluation in cranial defect model	[182]
DIW	Ionic + enzymatic + thermal crosslinking	Formation of porous/tube structure	Vascular tissue engineering	Repeated compression characteristics and anti-inflammatory response through multi-component bioink-based vascular design	Limited long-term in vivo evaluation	[183]
DIW	Thermal + GA crosslinking	Micron-scale designed spatial structure	Reduction of scar contracture	Vascular regeneration with dECM scaffolds, increased macrophage polarization toward M2 phenotype	Limited long-term clinical validation	[184]
FDM	PDA-assisted RGD immobilization	Orthogonal porosity structure	Improvement of osteogenic differentiation	Enhancing osteogenesis through improved nutrient transport and osteoblast attachment	Lack of in vivo validation	[185]
DLP	Photo-crosslinking	Orthogonally aligned fibrous stroma layer	Corneal regeneration	Promoting corneal regeneration through epithelial and stromal repair with enhanced transparency and nutrient transport	Reduced transparency at higher PEGDA content	[186]
DLP	Dual-network crosslinking	Collagen-based fibrous matrix with porous network and bioactive microenvironment	Wound healing	Accelerating wound healing through the promotion of cell proliferation, migration, and differentiation	Relatively low mechanical strength	[187]
DLP	Photo-crosslinking	Dual-layer scaffold mimicking natural bone/cartilage structure	Osteochondral defect reconstruction	Improves bone–cartilage integration by promoting cell attachment and proliferation through a microporous structure	Limited load-bearing capacity	[188]

## Data Availability

No new data were created or analyzed in this study.
